# High-moisture alfalfa (*Medicago sativa* L.) silage and the impact of alternative agricultural additives: industrial hemp (*Cannabis sativa* L.) and freeze-dried orange powder (*Citrus* × *sinensis*)

**DOI:** 10.3389/fpls.2026.1973950

**Published:** 2026-10-28

**Authors:** Jourdan E. Lakes, Gene L. Olson, Ying Wu, Huihua Ji, S. Ray Smith, Michael D. Flythe

**Affiliations:** 1 Forage-Animal Production Research Unit, United States Department of Agriculture (USDA) Agricultural Research Service, Lexington, KY, United States; 2 Kentucky Cooperative Extension Service, Department of Plant and Soil Sciences, Martin-Gatton College of Agriculture, Food and Environment, University of Kentucky, Lexington, KY, United States; 3 Kentucky Tobacco Research & Development Center, Martin-Gatton College of Agriculture, Food and Environment, University of Kentucky, Lexington, KY, United States

**Keywords:** alfalfa silage, HEMP, high-moisture, *Medicago sativa L*., orange powder, stored forage

## Abstract

**Introduction:**

The objective was to evaluate the effect of plant-derived additives from oranges (*Citrus* ***×*** *sinensis*) and industrial hemp (*Cannabis sativa* L.) on the composition and characteristics of direct-cut alfalfa (*Medicago sativa* L.) silage.

**Materials and methods:**

A 2^nd^ cutting of Ameristand 433T RR alfalfa was ensiled in two groups: unamended or 20 mL DI water added (WA). Each group had three treatments with two application rates each:: T1 – hemp crude oil extract (2 or 4 g kg^-1^), T2 – 2mm ground hemp hurd (5 or 10 g kg^-1^), and T3 – freeze-dried orange powder (40 or 60 g kg^-1^). The treated or control (unamended or WA; a subset was retained for pre-ensiling compositional analysis) material (~ 120 g bag^-1^) was sealed in vacuum bags and ensiled at 25 °C over 40 days. Post-ensiling, two sub samples were taken for spore-forming bacteria counts and fermentation analysis. The remainder of the post-ensiled material underwent compositional analysis.

**Results and discussion:**

The results indicate that addition of the freeze-dried orange powder facilitated a reduction in proteolysis, marked by a low pH and decreased ammonia. However, there were limited differences in lactic and acetic acids produced across the conditions. Propionic and butyric acids were consistently below the limits of quantification (< 1.0 mM). Addition of orange powder, particularly at 60 g kg^-1^, increased % DM but lowered % CP. There were inter-treatment variabilities, however the hemp treatments did not have a significant impact on silage quality. Additionally, there were no differences in spore-forming bacteria counts regardless of condition or treatment.

**Conclusion:**

High-moisture alfalfa is known to preserve poorly due to its high buffering capacity and low water-soluble carbohydrate content limiting the necessary rapid reduction in pH. Collectively, the data suggests that the use of freeze-dried orange powder, but not hemp crude oil or ground hurd, in direct-cut alfalfa appears to retain silage quality within an acceptable range for high-moisture legume silages.

## 1 Introduction

The fermentation, storage, and subsequent feedout of ensiled forages and crops is an important component of ruminant feeding systems. Silage is the feedstuff produced by microbial fermentations of crops and forages with moisture content usually greater than 50% (Undersander et al., 2011). Proper fermentation is essential and is impacted by several factors. For example, when the material is first cut, chopped, wilted, and packaged the microenvironment still contains oxygen whereby plant cells continue to respire, enzymes breakdown tissues, and problematic microorganisms persist, creating undesirable by-products and toxins, potentially impacting the quality of the fermentation (Muck, 1988). It is essential to move through this aerobic stage of ensiling as quickly as possible and into the fermentation stage whereby the microenvironment becomes anaerobic due to microbial respiration. In the fermentation stage of ensiling, the plant cells lyse releasing a slew of water-soluble carbohydrates (WSCs), which are quickly metabolized by lactic acid producing bacteria (LAB) subsequently lowering the pH of the silage (Kung et al., 2018; Muck, 1988). To a lesser extent, other fermentation acids and alcohols are produced at this stage (e.g., acetic, propionic, formic acids and ethanol) (Kung et al., 2018). The fermentation stage is critical as it actively inhibits the growth of harmful spoilage bacteria and fungi, allowing for proper preservation and storage of the ensiled material.

Not all forages and crops are ideal ensiling candidates due to factors such as sugar content and buffering capacity. For example, legumes, such as alfalfa (*Medicago sativa* L.), have a low sugar content and high buffering capacity, compared to cereal crops (e.g. – corn, *Zea mays* L.) and grasses (e.g. – perennial ryegrass, *Lolium perenne* L.), which makes them difficult to preserve as silage. Due to these factors, alfalfa has known vulnerability to harmful secondary clostridial fermentation and subsequent spoilage, especially when ensiled at higher moisture contents (> 70%) (Coblentz and Muck, 2012; Zhao et al., 2021). Researchers and producers have discovered ways to manage these risks largely through wilting the material prior to ensiling. However, additives (acids) (Ling et al., 2022), inoculants (LAB) (Zhao et al., 2021), and forage mixing (Ni et al., 2018; Zhang et al., 2017) have also proven viable methods to improve the silage quality of difficult-to-ensile crops and forages.

The objective of the present study was to determine if direct-cut (and direct-cut with water-added) alfalfa silage could be preserved through the application of one of three different additives (crude hemp extract oil, 2mm ground hemp hurd, and powderized freeze-dried orange slices) with two application rates per additive. The first additive, crude hemp oil extract, was previously identified by our group to contain cannabidiol, a known inhibitor of agriculturally relevant clostridia, including the silage spoilage bacterium, *Clostridium sporogenes* MD1 (Lakes et al., 2024). The second additive, (2mm) ground hemp hurd from a fiber cultivar Jin Ma, was chosen as a potential antimicrobial/absorbent material (Khan et al., 2015). The third additive, powderized freeze-dried oranges was selected for its sugar content, which is known to improve fermentation quality of silage as both a substrate source and natural acidulant (due to citric and ascorbic acid content), as well as the known antimicrobial content of two prominent volatile terpenes: limonene and α/β-pinene (Lakes et al., 2025; Lampitt and Baker, 1942; Li et al., 2020; Muck and O'Kiely, 1992; Zheng et al., 2017). Given that orange pulp has previously demonstrated promise as a silage additive of wilted alfalfa, the current study sought to investigate a different form of orange product (freeze-dried powder) to evaluate its efficacy under high-moisture ensiling conditions (Besharati et al., 2020). Additionally, orange products, such as those used here, should be relatively simple to obtain and augment (freeze-drying) as a byproduct of the orange industry.

## 2 Materials and methods

### 2.1 Reagents

Ethylene glycol (reagent grade) and hexanes (reagent grade) were purchased from Spectrum Chemical MFG Corp. (New Brunswick, NJ, USA). Standards of α/β-pinene, myrcene, linalool, (R)-limonene, β-caryophyllene, and terpinolene were purchased from Dr Ehrenstorfer LGC Standards (LGC Labor GmbH, Augsburg, Germany) for analysis of volatile compounds in orange powder. All standards were prepared in isopropanol (Fisher Scientific, Waltham, MA, USA), except for (R)-limonene and β-caryophyllene, which were prepared in methanol (Fisher Scientific). Volatile free acid mix reference material for high-performance liquid chromatography (HPLC) analysis was purchased from Millipore Sigma (Burlington, MA, USA), which contains the following reference acids: acetic, butyric, formic, heptanoic, hexanoic, isobutyric, isovaleric, 4-methylvaleric, propionic, and valeric acids.

### 2.2 Planting and harvesting conditions

Ameristand 433T RR (Round-up Ready™) alfalfa was planted on the University of Kentucky’s North Farm April of 2023. The stands were cut approximately 4 – 5x in any given year depending on annual rainfall, temperature (**Table 1**), and subsequent growth rates. For this experiment, a 2nd cutting of the alfalfa stand in plot 131RR was taken June 2025 using a forage harvester and approximately 75.7 L were collected of the cut alfalfa and transported to the Forage-Animal Production Research Unit laboratories on the University of Kentucky’s main campus for subsequent experimentation.

**Table 1:** Climate data for growing years 2023 – 2025.

Item^1^	Month of the year												Annual Average
	Jan	Feb	March	April	May	June*	July	Aug	Sept	Oct	Nov	Dec	
2023													
Temperature	6.1	8.3	8.8	14.1	18.3	21.9	25.7	24.6	21.9	15.9	9.2	6.6	15.1
Precipitation	5.1	3.3	3.6	2.0	2.0	5.8	4.3	2.0	0.8	1.8	1.5	2.0	2.8
2024													
Temperature	1.1	7.4	10.6	15.4	20.7	24.5	26.1	24.9	22.3	15.9	11.3	5.2	15.4
Precipitation	5.1	4.1	3.0	3.8	4.6	2.5	3.3	2.8	5.3	0.3	3.6	3.6	3.6
2025*													
Temperature	-1.8	4.1	10.8	14.6	17.1	23.8	26.4	23.5	21.3	14.5	7.7	2.8	13.7
Precipitation	2.8	6.1	3.3	8.9	6.1	4.8	4.1	0.5	2.8	6.6	2.0	2.3	4.3

^1^ Average temperature per month is given in degrees Celsius, and average precipitation per month is given in mm; data was obtained from the National Weather Service: https://www.weather.gov/wrh/Climate?wfo=lmk Accessed: April 24, 2026.

*The alfalfa used in the present study was harvested June, 2025.

### 2.3 Direct-cut alfalfa silage experiment

The alfalfa was chopped to ~2.5 cm pieces with scissors and ~120 g of material (79.6% moisture) was weighed and deposited into vacuum seal bags for a total of 42 mini-silage bags. Six bags were retained as controls, where one triplicate was prepared as unamended direct-cut silage controls and the other was amended with ~20 mL DI water to challenge the direct-cut silage further, as water addition to silages (e.g., wet silage) has been shown to favor clostridial fermentations (Kung et al., 2018). However, given that the material is ensiled directly (i.e., high-moisture), further water addition is expected to have limited effect(s) beyond impacts to dry matter percentage. The remaining bags were amended with either crude hemp oil (2 or 4 g kg^-1^; initial pH in water ~4.4), 2mm ground hemp hurd from cultivar Jin Ma described by Lakes et al. (2026) (5 or 10 g kg^-1^; initial pH in water ~6.0), or powderized freeze-dried orange slices (40 or 60 g kg^-1^; initial pH in water ~4.2). These treatments were repeated in triplicate with either added or no water added (~20 mL DI water). The ground hemp hurd and powderized freeze-dried orange slices were homogenized in the silage bags by vigorous mechanical shaking, whereas the crude hemp oil was applied in layers (i.e., layer of chopped alfalfa, layer of hemp oil, etc.; lasagna method) followed by kneading of the sealed bags to work in the oil further. All bags were sealed with a commercially purchased vacuum sealer (LEM MaxVac^®^ 250; West Chester, OH, USA) then incubated at 25 °C for 40 days in a standup incubator. Importantly, the bags were not laid on top of each other, but instead in a single layer (side-by-side) to avoid uneven heating during the ensiling process. Of note, while all treatments, conditions, and controls were repeated in triplicate, an n=3 is a small sample size and therefore the results of this study should be interpreted with this power limitation in mind. Additionally, initial compositional analysis, other than pH, was not performed for any of the additives (hemp-derived or orange powder), therefore their direct contributions to the ensiling process outside of pH could not be directly assessed.

### 2.4 Volatiles extraction and analysis

Volatile compounds from freeze-dried orange slices (purchased commercially from 52USA^®^, Felicific Inc. Queens, NY, USA) were extracted using a Clevenger apparatus (Wilmad-LabGlass™, Vineland, NJ, USA) setup. Briefly, a recirculating chiller (50:50 ethylene glycol: DI water) was set to -4 °C and the spoke of the apparatus was allowed to cool. Approximately 3 g of freeze-dried orange slice was added to a 300 mL long-necked round bottom flask and 150 mL Milli-Q^®^ water was added. After which the round bottom flask was affixed to the mouth of the apparatus, steadied with a clamp stand and the bottom of the flask was placed into a combo mantle heating element (Glas-Col, Terre Haute, IN, USA). The Clevenger arm was filled first with 2 mL of Milli-Q^®^ water then 1 mL of hexanes after which the chiller spoke was placed in the top opening of the apparatus and the heating element was set to 40%. The freeze-dried orange/water mixture was then boiled for 3h, after which the heating element was turned off, and the glassware was allowed to cool until it could be safely handled. The spoke was removed from the top of the apparatus, the hexane layer containing the extracted volatiles was collected with a long, glass Pasteur pipette and stored at -20 °C until analysis via gas chromatography – tandem mass spectrometry (GC-MS/MS) was performed.

An Agilent 7890B gas chromatograph with 7000C Triple Quadrupole mass spectrometer system coupled with a 7693 autosampler was used for volatile terpene analysis. Analytes were separated with a 30m × 250 µm × 0.25 µm GC column (Agilent DB-17 MS) at a flow rate of 1.2 mL min^-1^ using helium as a carrier gas. The column oven temperature was programmed as follows: initial temperature of 40 °C increased to 180 °C at a rate of 5 °C min^-1^, then ramped to 280 °C at a rate of 100 °C min^-1^, which was then held for 5 min. Injection port temperature was maintained at 250 °C and the sample was injected at a volume of 2.0 µL with a split ratio of 10:1. Total runtime was 36 min. Three technical replicates of the freeze-dried and powderized orange extract were analyzed. It should be noted that the terpene analysis was only performed on the raw freeze-dried orange powder and not on the fresh alfalfa itself or the post-ensiled material therefore their persistence throughout the ensiling process could not be verified.

### 2.5 Silage analysis

Three replicates of the freshly cut and chopped alfalfa (100 g each), as well as three replicates of water-added (WA) alfalfa (100 g each), were retained and stored at -20 °C for initial compositional analysis of the material. Following the completion of the 40-day ensiling period, the bags were opened and two subsamples were collected from each: 1 g sample for spore-forming microbial counts, and 10g sample for fermentation acids, ammonia, and pH analysis. After the subsamples were taken, the bags were resealed and stored immediately at -20 °C for post-ensiling compositional analysis. All compositional analyses of pre- and post-ensiled forage material were performed by a commercial forage testing laboratory, DairyOne, Inc. (Ithaca, NY, USA). Briefly, crude protein (CP) was analyzed using either a Leco TruMac N Macro or CN928 (Leco Corp., St. Joseph, MI, USA) according to the Association of Official Analytical Chemists (AOAC) protocol 990.03 (AOAC International, 2023). Acid detergent fiber (ADF) and amylase-treated neutral detergent fiber (aNDF) were analyzed using the ANKOM DELTA Digestion Unit (ANKOM Technology, Macedon, NY, USA) with solutions specified in AOAC 973.18 (AOAC International, 2023) and Van Soest et al. (1991), respectively. Dry matter (DM) analysis was performed by oven drying at 135 °C for 2 hours according to AOAC 930.15 (AOAC International, 2023). Non-fiber carbohydrates (NFC) were calculated using an equation reported by the National Research Council (2001) and total digestible nutrients (TDN) were calculated based on summative equations using chemical composition data and estimates of metabolic cost (Weiss et al., 1992).

The 10 g samples were transferred into individual 50 mL conical tubes, stored at -20 °C, and processed in batches of 6 to prevent further microbial action during the sample processing time. The fermentation acids (volatile fatty acids; VFAs) were extracted from the 10g of ensiled material following a protocol by Muck and O'Kiely (1992). Briefly, the material was placed in a sterile blender with 100 mL of MilliQ water and homogenized on the highest setting for 30 s. The homogenized sample was then filtered through 4 layers of sterile cheesecloth and centrifuged at 25,000 × g for 25 min at 4 °C (Sorvall Lynx 4000 centrifuge; Thermo Scientific; Waltham, MA, USA) with JA-14 rotor (Beckman Coulter; Brea, CA, USA). Two aliquots were taken from the supernatant for ammonia and VFA analysis, respectively, and were stored at -20 °C until the analysis was performed. The pH was measured from the sample liquid using a HI2209 Hanna Instruments^®^ pH meter (Woonsocket, RI, USA), and ammonia production was quantified using a phenolic acid/hypochlorite protocol originally developed by Chaney and Marbach (1962) and later modified by Cotta and Russell (1982) for cell culture.

VFA analysis was performed using the Vanquish Core HPLC system (Dionex Softron GmbH™, Germering, Germany) equipped with an Aminex HPX-87H column with BioRad Micro-Guard Cation H guard column set at 50 °C. The run was isocratic with a flow rate of 0.4 mL min^-1^ and total run time of 55 min; a 5 mM sulfuric acid mobile phase was used. Analytes were measured with a variable wavelength detector (210 nm, 20 Hz data collection rate; Dionex Softron GmbH™, Germering, Germany) and a refractive index detector (2 Hz, 3 s response rate, set to 35 °C; Dionex Softron GmbH™, Germering, Germany).

### 2.6 Culture media composition

Spore-forming microbes were cultured in basal medium based on Chen and Russell (1989), the composition of which is detailed in **Supplementary Table 1**. After the components were mixed in 1 L of DI water, the pH of the broth was adjusted to 6.5 using 5 M NaOH, then autoclaved at 121 °C for 20 min to remove O_2_ and finally cooled under CO_2_. After cooling, 4.0 g Na_2_CO_3_ buffer was added and allowed to dissolve fully. The broth was then tubed anaerobically using Hungate tubes, butyl rubber stoppers, and hard plastic caps after which the media was autoclaved again for sterility.

### 2.7 Spore-forming bacterial counts

After the 40-day ensiling process was complete, the bags were opened and 1 g of ensiled material was removed from each bag for spore-forming microbial counts. Briefly, the subsampled material was placed in a 15 mL conical tube and 9 mL of basal medium (described above) was added. The tubes were sealed, mechanically shaken, and vortexed for ~30 s each. After mixing, 1 mL was extracted from mid-depth and inoculated into a fresh tube containing 9 mL of basal medium. The tubes were then mixed and serially diluted 10% v/v 12x into basal media. All tubes were then pasteurized for 30 min at ~ 80 °C. The pasteurized tubes were then placed in a 39 °C (bovine ruminant body temperature) standup incubator and growth results taken after 15 days to allow ample time for spore germination and growth.

### 2.8 Statistical analysis

All experiments and treatments were performed in triplicate. All statistical analyses were performed using OriginPro^®^ software (v10.2.0.196). Normality of data was determined using a Shapiro-Wilk test, and homogeneity of variances was determined using Brown-Forsythe test. Statistically significant main effects and interactions of the silage chemical composition and fermentation characteristics were determined using three-way ANOVA (accounting for water application, treatment, and application rate) and Tukey’s *post-hoc* test (p < 0.05). Significant differences between unamended and water-added day 0 nutritive analysis (chemical composition; **Table 2**) were determined using a two-sample t-test (p < 0.05).

**Table 2:** Chemical composition of fresh alfalfa.

Item^1^	Alfalfa	
	Unamended	Water-added
% DM*	20.4 (0.17)	17.1 (0.10)
% CP	21.1 (0.87)	21.2 (0.50)
% ADF	29.6 (1.88)	28.9 (2.22)
% aNDF	35.3 (2.37)	38.1 (1.85)
% NFC	30.5 (1.70)	27.5 (2.40)
% TDN	62.3 (0.58)	61.7 (0.58)

Values in parentheses represent standard deviation.

^1^ DM, dry matter; CP, crude protein; ADF, acid detergent fiber; aNDF, neutral detergent fiber; NFC, non-fiber carbohydrates; TDN, total digestible nutrients; Significant differences across a row are indicated by the presence of an asterisk (p < 0.0001).

## 3 Results

### 3.1 Chemical composition of fresh direct-cut alfalfa

The results for the chemical characterization of the pre-ensiled alfalfa material are summarized in **Table 2**. The percent DM, CP, ADF, aNDF, NFC, and TDN of pre-ensiled direct-cut alfalfa were 20.4 (moisture of 79.6%), 21.1, 29.6, 35.3, 30.5, and 62.3, respectively. The chemical composition of the direct-cut alfalfa was also measured for samples amended with ~20 mL DI water prior to ensiling; percent DM, CP, ADF, aNDF, NFC, and TDN were 17.1 (moisture of 82.9%), 21.2, 28.9, 38.1, 27.5, and 61.7, respectively (**Table 2**). Between the unamended and water-added (WA) alfalfa samples, only % DM (and moisture) was significantly different, as expected given the addition of 20 mL DI water.

### 3.2 Chemical composition of alfalfa silage

The addition of water predictably decreased the % DM content and there was a significant interaction identified between water addition and treatment (p = 0.008; **Table 3**). Indeed, for all treatments, the means of the water added (WA) versus unamended groups were significantly different from each other (p < 0.0001). A significant interaction between treatment group and application rate was also identified (p = 0.001; **Table 3**); namely, as application rate increased, % DM increased. This interaction was particularly noticeable in the ground hemp hurd (T2–1 and T2–2 unamended and WA; p = 0.02) and orange powder treatments (T3-1/T3–2 unamended and WA; p < 0.0001) more so than the control and hemp oil (T1-1/T1–2 unamended and WA). All treatments had largely comparable % CP values, however there was a significant interaction between water addition and treatment present (p=0.002; **Table 3**) in the control where WA decreased % CP (p < 0.0001), but such an interaction was not significant for the other treatments (p > 0.90). Similarly, a significant interaction between treatment and application rate (p = 0.014) was noted in T3-1/T3-2 (unamended and WA) where an increase in application rate decreased % CP (p = 0.008); this interaction was not significant in either T1-1/T1-2 (unamended and WA; p = 0.55) or T2-1/T2-2 (unamended and WA; p = 0.78). The significant interaction between treatment and application rate persisted in % ADF (p = 0.0008), aNDF (p = 0.003), NFC (p = 0.002), and TDN values (p = 0.035), whereas the significant interaction between water addition and treatment was lost (**Table 3**). For both % ADF and aNDF, the interaction between treatment and application was significant in alfalfa silage treated with hemp oil (T1–1 and T1-2, unamended and WA) where, as the application rate increased, the % ADF and aNDF decreased (p = 0.0001 and 0.0004, respectively). While there was not a significant interaction between water addition and treatment for % aNDF (p = 0.72), there was a main effect difference identified with water addition (p = 0.002; **Table 3**) where WA increased % aNDF. As application rate increased in T1–1 and T1-2 (unamended and WA), % NFC and TDN increased (p = 0.0002 and 0.003, respectively). Notably, in % NFC there was a slight though not significant increase in the orange powder treatments (unamended and WA) as application rate increased (p = 0.05). Lastly, as noted for % aNDF, while there was not a significant interaction between water addition and treatment, there was a significant main effect with water addition for both % NFC and TDN, where WA decreased both metrics (p = 0.002 and 0.007, respectively; **Table 3**).

**Table 3:** Chemical composition of direct-cut alfalfa silage either treated or untreated with plant components.

Treatments^1^	Chemical composition^2^					
	% DM	% CP	% ADF	% aNDF	% NFC	% TDN
Unamended						
Control	19.1 (0.12)	23.3 (0.25)	32.5 (0.85)	37.4 (0.97)	26.1 (1.17)	62.0 (0.00)
T1-1	19.3 (0.12)	22.2 (0.59)	34.8 (1.08)	40.9 (1.34)	23.8 (0.79)	61.0 (0.00)
T1-2	19.4 (0.29)	22.8 (0.40)	30.4 (1.22)	35.5 (0.68)	28.6 (0.64)	62.3 (0.58)
T2-1	19.1 (0.17)	22.4 (0.82)	32.2 (0.95)	37.8 (1.68)	26.8 (1.32)	61.7 (0.58)
T2-2	19.5 (0.15)	22.5 (0.89)	32.3 (2.26)	38.2 (3.84)	26.2 (3.08)	61.7 (1.16)
T3-1	20.9 (0.40)	20.9 (0.15)	29.6 (1.42)	36.1 (0.87)	30.0 (0.74)	62.3 (0.58)
T3-2	21.9 (0.15)	20.1 (0.15)	28.1 (2.05)	33.8 (1.86)	32.9 (1.85)	62.7 (0.58)
Water-added (WA)						
Control WA	16.5 (0.46)	22.0 (0.55)	32.7 (1.91)	40.5 (3.46)	24.4 (2.86)	61.0 (1.00)
T1–1 WA	15.9 (0.25)	22.4 (0.17)	34.9 (1.34)	41.7 (0.91)	22.8 (0.90)	60.7 (0.58)
T1–2 WA	16.3 (0.12)	22.7 (0.64)	31.6 (0.67)	36.9 (0.99)	27.3 (0.45)	62.0 (0.00)
T2–1 WA	16.5 (0.20)	22.6 (0.63)	31.5 (0.67)	38.6 (2.46)	25.6 (2.05)	61.3 (0.58)
T2–2 WA	17.1 (0.15)	21.8 (0.21)	34.5 (0.95)	41.8 (0.06)	23.3 (0.29)	61.0 (0.00)
T3–1 WA	17.8 (0.50)	21.3 (0.26)	30.8 (2.44)	37.7 (3.40)	27.9 (3.15)	62.0 (1.00)
T3–2 WA	18.8 (0.50)	20.2 (0.15)	29.2 (1.17)	36.1 (2.04)	30.6 (2.14)	62.3 (0.58)
ANOVA	p-values					
Water addition (A)	**<0.0001**	**0.036**	0.12	**0.002**	**0.002**	**0.007**
Treatment (B)	**<0.0001**	**<0.0001**	**<0.0001**	**0.003**	**<0.0001**	**0.004**
Application rate (C)	**<0.0001**	0.15	**0.028**	**0.041**	**0.009**	0.053
A × B	**0.008**	**0.002**	0.90	0.72	0.91	0.55
A × C	0.48	0.19	0.28	0.43	0.59	0.83
B × C	**0.001**	**0.014**	**0.0008**	**0.003**	**0.002**	**0.035**
A × B × C	0.86	0.64	0.57	0.88	0.94	0.99

Values in parentheses represent standard deviation; statistically significant interactions are boldened.

^1^ WA, water-added; T1-1 (WA), hemp crude oil extract 2g kg^-1^; T1-2 (WA), hemp crude oil extract 4g kg^-1^; T2-1 (WA), ground hemp hurd 5g kg^-1^; T2-2 (WA), ground hemp hurd 10g kg^-1^; T3-1 (WA), orange powder 40g kg^-1^; T3-2 (WA), orange powder 60g kg^-1.^

^2^ DM, dry matter; CP, crude protein; ADF, acid detergent fiber; aNDF, neutral detergent fiber; NFC, non-fiber carbohydrates; TDN, total digestible nutrients.

### 3.3 Fermentation quality and spore-forming microbe counts of alfalfa silage

The fermentation characteristics of the untreated and treated alfalfa silage are summarized in **Table 4**. The pH values of the silage controls (with or without water-added; WA) were 5.0 with subsequent ammonia concentrations of 6.9 (WA) or 7.3 (unamended) mM, respectively. There were no significant interactions affecting pH identified. However, significant water addition, resulting in lower pH, and treatment main effects were observed (p = 0.0001 and < 0.0001, respectively). Within the treatments, orange powder (T3–1 and T3–2 unamended and WA) had significantly lower pH than control, hemp oil (T1–1 and T1–2 unamended and WA), and ground hurd (T2–1 and T2–2 unamended and WA; p < 0.0001). Similarly to pH, there were no significant interactions identified affecting ammonia concentrations (**Table 4**). However, a treatment main effect was identified (p < 0.0001) as in the pH data, where T3–1 and T3-2 (unamended and WA) had significantly lower ammonia concentrations than control, hemp oil, and ground hurd (p < 0.0001). Unlike the pH data, ammonia concentration of alfalfa silage treated with ground hurd (T2–1 and T2–2 unamended and WA) were also significantly different (lower) than control (p = 0.02). However, despite the marked reduction in ammonia concentration, the number of spore-forming amino acid fermenting bacteria (selected for by the growth medium) in the alfalfa silages did not differ regardless of treatment or application rate (10^2–^10^3^ cells; **Supplementary Table 2**).

**Table 4:** Fermentation characteristics of direct-cut alfalfa silage either treated or untreated with plant components.

Treatments^1^	Fermentation characteristics							
	pH	Ammonia	Lactic acid	Acetic acid	Propionic acid	Butyric acid	Ethanol	Lactic acid/Acetic acid
		mM^2^						
Unamended								
Control	5.0 (0.06)	7.3 (0.46)	11.1 (0.94)	5.4 (0.16)	< LOQ	< LOQ	3.9 (0.18)	2.1 (0.21)
T1-1	5.1 (0.03)	6.4 (0.59)	8.4 (1.10)	4.5 (0.41)	< LOQ	< LOQ	3.4 (0.59)	1.9 (0.07)
T1-2	5.4 (0.08)	7.6 (0.95)	8.2 (0.42)	5.3 (0.58)	< LOQ	< LOQ	3.6 (0.19)	1.5 (0.08)
T2-1	5.1 (0.03)	6.9 (0.67)	9.4 (1.61)	4.9 (0.57)	< LOQ	< LOQ	3.8 (0.64)	1.9 (0.11)
T2-2	5.2 (0.06)	6.8 (0.76)	8.8 (1.22)	4.5 (0.63)	< LOQ	< LOQ	3.4 (0.38)	1.9 (0.07)
T3-1	4.7 (0.16)	4.9 (0.73)	10.3 (0.72)	4.9 (0.16)	< LOQ	< LOQ	3.3 (0.26)	2.1 (0.11)
T3-2	4.6 (0.11)	4.5 (0.26)	10.2 (0.86)	5.4 (0.65)	< LOQ	< LOQ	3.4 (0.08)	1.9 (0.08)
Water-added (WA)								
Control WA	5.0 (0.26)	6.9 (1.45)	9.0 (1.58)	5.8 (1.34)	< LOQ	< LOQ	3.8 (1.25)	1.6 (0.59)
T1–1 WA	5.0 (0.19)	6.9 (1.05)	9.3 (2.06)	7.4 (1.70)	< LOQ	< LOQ	3.9 (0.62)	1.4 (0.53)
T1–2 WA	4.9 (0.05)	6.6 (0.55)	10.5 (0.67)	5.9 (0.53)	< LOQ	< LOQ	2.9 (0.20)	1.8 (0.15)
T2–1 WA	5.0 (0.20)	5.8 (1.15)	9.6 (0.50)	5.0 (0.76)	< LOQ	< LOQ	3.6 (0.53)	2.0 (0.35)
T2–2 WA	4.9 (0.05)	5.6 (0.29)	9.8 (0.42)	4.4 (0.30)	< LOQ	< LOQ	3.2 (0.10)	2.2 (0.14)
T3–1 WA	4.5 (0.03)	4.7 (0.48)	10.6 (0.37)	6.2 (0.37)	< LOQ	< LOQ	4.4 (0.33)	1.7 (0.04)
T3–2 WA	4.5 (0.13)	4.4 (0.22)	10.2 (1.23)	6.8 (0.95)	< LOQ	< LOQ	4.2 (0.33)	1.5 (0.28)
ANOVA	p-values							
Water addition (A)	**0.0001**	0.05	0.83	**0.0007**			0.52	**0.03**
Treatment (B)	**<0.0001**	**<0.0001**	**0.06**	**0.01**			0.21	**0.03**
Application rate (C)	0.89	0.89	0.99	0.76			0.22	0.95
A × B	0.13	0.45	**0.003**	**0.03**			0.06	0.06
A × C	0.15	0.38	0.46	0.22			0.26	0.15
B × C	0.78	0.68	0.87	0.45			0.80	0.57
A × B × C	0.12	0.57	0.76	0.24			0.51	0.36

LOQ, limit of quantification (< 1.0 mM); values in parentheses represent standard deviation; statistically significant interactions are boldened.

^1^ WA, water-added; T1-1 (WA), hemp crude oil extract 2g kg^-1^; T1-2 (WA), hemp crude oil extract 4g kg^-1^; T2-1 (WA), ground hemp hurd 5g kg^-1^; T2-2 (WA), ground hemp hurd 10g kg^-1^; T3-1 (WA), orange powder 40g kg^-1^; T3-2 (WA), orange powder 60g kg^-1.^

^2^ Fermentation acid concentrations were calculated on a %DM-basis as well and are presented in **Supplementary Table 3**.

A significant interaction between water addition and treatment was noted for both lactic and acetic acid concentrations in the silage (p = 0.003 and 0.03, respectively; **Table 4**). WA decreased lactic acid in the control (p = 0.009) and significantly increased the acetic acid in both the hemp oil (T1–1 and T1-2) and orange powder (T3–1 and T3-2) treatments (p = 0.001 and 0.02, respectively). Lastly, the lactic/acetic acid (LA/AA) ratio was largely unchanged for all controls and treatments. However, water addition (p = 0.03) and treatment (p = 0.03) main effects were observed, where the former resulted in a minor decrease in LA/AA when water was added, and the latter was only significant between hemp oil and ground hurd treatments (p = 0.003) with hemp oil generally having a lower LA/AA than ground hurd. In the present study, the undesirable propionic and butyric acids were below the limits of quantification (LOQ: < 1.0 mM) for all conditions tested (**Table 4**). In addition to the organic acids found in silage, we also quantified ethanol, which was detectable in all controls and treatments, though the concentrations were low (< 4.5 mM), and there were no significant main effects or interactions identified. Lastly, the terpene analysis of the freeze-dried orange powder used in this study revealed that limonene was the most abundant followed by β-myrcene and linalool: 331.64, 8.56, and 5.24 µg mL^-1^, respectively (**Table 5**). α/β-pinene, terpinolene, and β-caryophyllene were also present but at lower concentrations.

**Table 5:** Volatile terpene content of freeze-dried orange slices.

Volatile orange compounds	µg mL^-1^
α-pinene	3.46 (0.07)
β-pinene	2.97 (0.03)
β-myrcene	8.56 (0.19)
Limonene	331.64 (3.35)
Terpinolene	1.25 (0.01)
Linalool	5.24 (0.08)
β-caryophyllene	1.64 (0.01)

Values in parentheses represent standard deviation.

## 4 Discussion

The alfalfa used in this study had characteristically low % DM and high % CP content (Gordon et al., 1961; Li et al., 2020; Zheng et al., 2017). Post-ensiling characteristics of the control alfalfa silage were also consistent with previous reports utilizing direct-cut alfalfa, e.g., % DM 19.1 and % CP 23.3 (**Table 3**) (Gordon et al., 1961; Li et al., 2020; Ohshima et al., 1997). Treatment with orange powder increased % DM and lowered % CP, particularly as the application rate increased. This observation could be due to a dilution effect caused by the addition of a low protein – high carbohydrate substance (e.g., orange powder) that increases % DM without subsequent % CP increases (Vidotti et al., 2003). Despite this, a previous report by the National Research Council (2001) concluded that alfalfa silage was an excellent forage defined as containing % CP levels of 20 – 22, indicating that while the addition of the orange powder lowered % CP in alfalfa silage, the CP values reported in this study remained within range. Similarly, treatment T3-2 (orange powder, application rate 60 g kg^-1^) had reduced % aNDF (33.8%; **Table 3**). The same report by the National Research Council (2001) concluded that excellent alfalfa silage was also defined as containing % NDF 35 – 40. Importantly, from this criterion, the addition of freeze-dried orange powder at a rate of 60 g kg^-1^, without addition of water, lowers the % NDF out of the identified target range, while the 40 g kg^-1^ rate does not regardless of water addition (36.1 and 37.7%, unamended and WA, respectively). However, lower % NDF in silage is generally desirable as high NDF values tend to decrease palatability and intake (Shi et al., 2023).

pH is one of the key indicators of successful ensiling, which is largely influenced by two primary factors: organic acids produced by the fermentation process, and the forage material’s buffering capacity, which generally resists changes to pH (Muck, 1988). The pH values of the silage controls (unamended and WA) were predictably higher (5.0) given the lack of additives, low % DM, and alfalfa’s known high buffering capacity (**Table 4**) (Stepanova and Volovik, 2021). pH values were largely unaffected by the hemp treatments but were impacted by the addition of orange powder; specifically, orange powder addition lowered pH values (≤ 4.7). Indeed, the addition of sugar substrates has been shown to lower pH of alfalfa silage (Gao et al., 2021; Wang et al., 2020). Generally, a pH of 4.2 is considered benchmark for well-fermented high moisture silage (Edwards and McDonald, 1978), however, pHs ranging from 4.3 – 5.0 are considered common for acceptable legume silages (He et al., 2020). With these pH values as guidance, many of the alfalfa silages produced in the present study were within the boundaries of acceptable despite not reaching the pH 4.2 target. However, there were treatments/application rates that resulted in pH values > 5.0, such as hemp oil and hemp hurd treated silages (both application rates) that did not have additional water added (**Table 4**).

Importantly, alfalfa has known vulnerability to harmful secondary clostridial fermentation and subsequent spoilage, especially when ensiled at higher moisture content (> 70%) (Coblentz and Muck, 2012; Zhao et al., 2021; Zheng et al., 2018). Proteolytic activity by Clostridia results in the breakdown of proteins in forages, decreasing their nutritive value and creating byproducts (e.g., ammonia) that resist crucial acidification (Coblentz and Muck, 2012; Li et al., 2020). Predictably, the greatest ammonia concentrations were observed when alfalfa was ensiled without treatment intervention (e.g., unamended or WA control; ammonia ≥ 6.9 mM). As with the previous results, most hemp treatments did not have a significant impact on ammonia production whereas the addition of orange powder reduced ammonia concentrations. However, it should be noted that while the ammonia reduction was not as significant as the orange powder treatments, that treatment with ground hemp hurd also significantly reduced ammonia concentration in alfalfa silage. These results demonstrate that the addition of freeze-dried orange powder in particular, was able to significantly reduce the production of ammonia in high-moisture alfalfa silage.

Previous work from our group and others demonstrated that either individual phytochemicals or essential oils containing many such compounds (some shared between citrus fruits and hemp; e.g., limonene and pinene) are known to be antimicrobial against livestock pathogens such as *Fusobacterium necrophorum* and *Clostridium perfringens* (Lakes et al., 2025; Ouwehand et al., 2010). Our results indicated the presence of both antimicrobial phytochemicals, limonene and (α/β) pinene, in the orange powder material (**Table 5**), among others (Albayrak et al., 2023; Dahham et al., 2015; Liu et al., 2020). For these reasons, it was hypothesized that the addition of orange powder may control clostridial growth in high-moisture silages at risk of these harmful fermentations. However, despite the reduction in ammonia (a common indicator of clostridial fermentation, i.e., proteolysis) observed in the ensiled alfalfa treated with orange powder in particular, the data also demonstrated a lack of growth effect on spore-forming amino acid fermenting bacteria (such as the Clostridia). These results indicated that the reduction in ammonia in the orange powder treatments was not necessarily due to growth inhibition of ammonia-producing spore-forming bacteria selected by the culture medium. This latter result is not surprising. It was previously shown that the silage contaminant *Clostridium sporogenes* MD1 exhibited uncoupled metabolism and growth (Flythe and Russell, 2005). Uncoupled catabolism and anabolism are not uncommon among bacteria (Russell, 2007). Thus, a partial inhibition of ammonia production will not necessarily decrease growth rate or the number of viable bacteria.

Lactic acid is largely produced through the metabolism of LAB, which is a critical factor in the lowering of pH in ensiled forages (Muck, 1988). However, the data did not indicate a change in lactic acid production within the alfalfa silage under any of the treatment conditions or application rates. Therefore, it is unlikely that the orange powder additive served as a meaningful additional substrate source (e.g., sugar) for the LAB during the ensiling process under the conditions tested. However, it should be noted that alternative to orange powder addition lowering pH and ammonia concentrations through action as a sugar substrate or source of antimicrobial compounds, respectively, it could have lowered pH through its intrinsic properties as an acidulant (Lampitt and Baker, 1942). Indeed, we determined that the freeze-dried orange powder results in a pH of ~4.2 in DI water at the application rates used in the present study, which could provide support for this hypothesis. However, it was also determined that hemp oil has a pH of ~4.4 in DI water and the data did not reveal similar decreases in the pH of alfalfa silage as seen in those silages treated with orange powder. Therefore, it is possible that the orange powder additive exerts its impact on high-moisture alfalfa silage under a different mechanism or combination thereof.

Production of acetic acid can lower silage pH as well, albeit not as significantly as lactic acid, given that it is a weaker acid (Danner et al., 2003). However, the acetic acid concentrations reported in the present study did not exceed 7.4 mM and, similarly to the lactic acid data, were largely unchanged, despite WA slightly increasing the concentration in hemp oil and orange powder treatments. Lastly, in silages with good fermentation quality, the content of undesirable acids such as propionic and butyric acid should be as low as possible (Kung et al., 2018). Despite the limited changes in lactic and acetic acid concentrations and the high-moisture of the ensiled material, the propionic and butyric acid concentrations remained below LOQ. Given that there were no significant differences in organic acids to account for the changes in pH, primarily in the orange powder treatments, it is more likely that the significant reduction in ammonia (a basic compound) was responsible for the subsequent acidification observed.

In conclusion, these data suggest that the application of freeze-dried orange powder facilitates a reduction in proteolysis determined via reductions in ammonia concentration, though the mechanism(s) of this activity are presently unclear given the limited measurements of the current study. Importantly, the chemical composition (nutritive analysis) of this silage appears to be retained within an acceptable range for high-moisture legume silages. However, the effects of orange powder application should be investigated further under alternative ensiling (e.g., wilted) and treatment (e.g., fresh orange waste versus freeze-dried) conditions.

## Data Availability

The original contributions presented in the study are included in the article/**Supplementary Material**. Further inquiries can be directed to the corresponding author.
