# Supplementary material for: High-moisture alfalfa (Medicago sativa L.) silage and the impact of alternative agricultural additives: industrial hemp (Cannabis sativa L.) and freeze-dried orange powder (Citrus × sinensis)
Source: Front Plant Sci. 2026 Oct 28;17:1973950. doi: 10.3389/fpls.2026.1973950 (PMC13751355; doi:10.3389/fpls.2026.1973950)
Supplement: Supplementary file 1 [file Table1.docx]

Supplementary Material

**Supplementary Table 1**. Culture medium composition

| Culture Medium | Composition (per 1L) |
| --- | --- |
| Basal Medium (BM)^1^ | - 15 g tryptone peptone - 240 mg KH_2_PO_4_ - 240 mg K_2_HPO_4_ - 480 mg NaCl - 516 mg Na_2_SO_4_ - 100 mg MgSO_4_ • 7H_2_O - 64 mg CaCl_2_ • 2H_2_O - 1 mg pyridoxamine 2HCl - 2 mg thiamine HCl - 2 mg nicotinamide - 1 mg lipoic acid - 2 mg riboflavin - 1 mg pyridoxal HCl - 0.05 mg folic acid - 0.1 mg p-aminobenzoic acid - 0.05 mg cobalamin - 0.05 mg biotin - 1 mg pyridoxine - 2.5 mg Na_4_EDTA - 2 mg CaD pantothenate - 1 mg MnCl_2_ • 4H_2_O - 0.01 mg NiCl_2_ • 6H_2_O - 0.05 mg ZnSO_4_ • 7H_2_O - 1 mg FeSO_4_ • 7H_2_O - 0.1 mg CoCl_2_ • 6H_2_O - 0.1 mg H_3_BO_3_ - 5 μg CuCl_2_ • 2H_2_O - 0.015 mg NaMoO_4_ • 2H_2_O - 600 mg cysteine HCl |

^1^ BM was based on Chen & Russell (1989) for the growth of amino acid-fermenting (i.e., ammonia-producing) bacteria

| **Supplementary Table 2.** Spore-former counts of direct-cut alfalfa silage | | | |
| --- | --- | --- | --- |
| Treatments^1^ | Spore Growth |  |  |
| Unamended |  |  |  |
| Control | 10^2^ |  |  |
| T1-1 | 10^2^ |  |  |
| T1-2 | 10^2^ |  |  |
| T2-1 | 10^2^ |  |  |
| T2-2 | 10^2^ |  |  |
| T3-1 | 10^2^ |  |  |
| T3-2 | 10^2^ |  |  |
| Water-added (WA) |  |  |  |
| Control WA | 10^2^ |  |  |
| T1-1 WA | 10^2^ |  |  |
| T1-2 WA | 10^2^ |  |  |
| T2-1 WA | 10^2^ |  |  |
| T2-2 WA | 10^3^ |  |  |
| T3-1 WA | 10^2^ |  |  |
| T3-2 WA | 10^2^ |  |  |
| ^1^ WA = water-added; T1-1 (WA) = hemp crude oil extract 2g kg^-1^; T1-2 (WA) = hemp crude oil extract 4g kg^-1^; T2-1 (WA) = ground hemp hurd 5g kg^-1^; T2-2 (WA) = ground hemp hurd 10g kg^-1^; T3-1 (WA) = orange powder 40g kg^-1^; T3-2 (WA) = orange powder 60g kg^-1^ | | | |
|  |  |  |  |
|  |  |  |  |
|  |  |  |  |

| **Supplementary Table 3.** Fermentation characteristics of direct-cut alfalfa silage either treated or untreated with plant components on a % dry matter (DM)-basis | | | | | | |
| --- | --- | --- | --- | --- | --- | --- |
|  |  |  |  |  |  |  |
|  | Fermentation characteristics | | |  |  |  |
|  | Lactic acid | Acetic acid | Propionic acid | Butyric acid | Ethanol | Lactic acid/Acetic acid |
| Treatments^1^ | % DM-basis | | | | |  |
| Unamended |  |  |  |  |  |  |
| Control | 0.52 (0.04) | 0.17 (0.00) | < LOQ | < LOQ | 0.09 (0.00) | 3.1 (0.32) |
| T1-1 | 0.39 (0.05) | 0.14 (0.01) | < LOQ | < LOQ | 0.08 (0.01) | 2.8 (0.11) |
| T1-2 | 0.38 (0.01) | 0.16 (0.02) | < LOQ | < LOQ | 0.09 (0.00) | 2.3 (0.13) |
| T2-1 | 0.44 (0.07) | 0.16 (0.02) | < LOQ | < LOQ | 0.09 (0.02) | 2.9 (0.17) |
| T2-2 | 0.41 (0.06) | 0.14 (0.02) | < LOQ | < LOQ | 0.08 (0.01) | 2.9 (0.11) |
| T3-1 | 0.44 (0.03) | 0.14 (0.01) | < LOQ | < LOQ | 0.07 (0.00) | 3.1 (0.17) |
| T3-2 | 0.42 (0.04) | 0.15 (0.02) | < LOQ | < LOQ | 0.07 (0.00) | 2.8 (0.12) |
| Water-added (WA) |  |  |  |  |  |  |
| Control WA | 0.49 (0.07) | 0.21 (0.05) | < LOQ | < LOQ | 0.11 (0.04) | 2.5 (0.89) |
| T1-1 WA | 0.53 (0.11) | 0.28 (0.07) | < LOQ | < LOQ | 0.11 (0.02) | 2.0 (0.80) |
| T1-2 WA | 0.58 (0.04) | 0.22 (0.02) | < LOQ | < LOQ | 0.08 (0.01) | 2.7 (0.22) |
| T2-1 WA | 0.52 (0.02) | 0.18 (0.03) | < LOQ | < LOQ | 0.10 (0.01) | 3.0 (0.52) |
| T2-2 WA | 0.52 (0.02) | 0.15 (0.01) | < LOQ | < LOQ | 0.09 (0.00) | 3.4 (0.21) |
| T3-1 WA | 0.53 (0.03) | 0.21 (0.02) | < LOQ | < LOQ | 0.11 (0.01) | 2.5 (0.06) |
| T3-2 WA | 0.49 (0.07) | 0.22 (0.03) | < LOQ | < LOQ | 0.10 (0.01) | 2.3 (0.42) |
| Values in parentheses represent standard deviation | | | | | | |
| ^1^ WA = water-added; T1-1 (WA) = hemp crude oil extract 2g kg^-1^; T1-2 (WA) = hemp crude oil extract 4g kg^-1^; T2-1 (WA) = ground hemp hurd 5g kg^-1^; T2-2 (WA) = ground hemp hurd 10g kg^-1^; T3-1 (WA) = orange powder 40g kg^-1^; T3-2 (WA) = orange powder 60g kg^-1^ | | | | | | |
|  |  |  |  |  |  |  |
|  |  |  |  |  |  |  |
|  |  |  |  |  |  |  |
